# Desmosterol: A natural product derived from macroalgae modulates inflammatory response and oxidative stress pathways in intestinal epithelial cells

**DOI:** 10.3389/fimmu.2022.1101643

**Published:** 2023-01-04

**Authors:** Huan Qu, Qiufang Zong, Ping Hu, Zhaojian Li, Haifei Wang, Shenglong Wu, Hao-Yu Liu, Wenbin Bao, Demin Cai

**Affiliations:** ^1^ College of Animal Science and Technology, Yangzhou University, Yangzhou, China; ^2^ Joint International Research Laboratory of Agriculture & Agri-Product Safety, Yangzhou University, Yangzhou, Jiangsu, China

**Keywords:** desmosterol, algae extracts, inflammatory response, oxidative stress, RORγ

## Abstract

The serum level of cholesterol and its biosynthetic intermediates are critical indicators to access metabolism-related disorders in humans and animals. However, the molecular actions of these intermediates on gene functions and regulation remained elusive. Here, we show that desmosterol (DES) is the most abundant intermediate involved in cholesterol biosynthesis and is highly enriched in red/brown algae. It exerts a pivotal role in modulating core genes involved in oxidative stress and inflammatory response processes in the ileum epithelial cells (IPI-2I). We observed that the DES extracted from red algae did not affect IPI-2I cell growth or survival. A transcriptomic measurement revealed that the genes enrolled in the oxidative process and cholesterol homeostasis pathway were significantly down-regulated by DES treatment. Consistent with this notion, cellular reactive oxygen species (ROS) levels were markedly decreased in response to DES treatment. In contrast, key inflammatory genes including *IL-6*, *TNF-α*, and *IFN-γ* were remarkably upregulated in the RNA-seq analysis, as further confirmed by qRT-PCR. Given that DES is a specific agonist of nuclear receptor RORγ, we also found that DES caused the elevated expression of RORγ at mRNA and protein levels, suggesting it is a potential mediator under DES administration. Together, these results underscore the vital physiological actions of DES in inflammatory and oxidative processes possibly *via* RORγ, and may be helpful in anti-oxidation treatment and immunotherapy in the future.

## 1 Introduction

Red algae are ubiquitous marine macroalgae that have developed bioactive plasticity and compound diversity. It is known that desmosterol (DES) is a dominating sterol in red macroalgae with 87-93% of total sterol contents (187-337 μg/g dry weight) (1). Sterols are fundamental components of cell membranes’ phospholipid bilayer that include molecules (such as cholesterol and DES) and are responsible for structural and functional roles. DES, a biosynthetic cholesterol intermediate of the Bloch pathway, plays essential roles in some specific circumstances. DES may contribute to cell membrane fluidity and promote sperm maturation. For instance, DES accounts for 25% of sperm sterol in males (2), and the proportion reaches 60% in several animals (3). Furthermore, DES maintains cell proliferation and survival with or without cholesterol supplementation in *Dhcr24*-defective J774 cells (4). Interestingly, DES acts as a precursor of steroidogenesis even better than cholesterol (4). Additionally, biosynthetic DES is an emergent regulator of macrophages during the process of lipid overload (5). Although a number of biological functions have been reported, the molecular actions of DES on gene functions and its direct regulation have remained elusive.

Cholesterol deposition or prolongation facilitates a progressive inflammatory response and immune response associated with disease development (6). Specifically, the innate immune system amplifies the inflammatory signal by modulating cholesterol homeostasis (6). Notably, the balance of cholesterol metabolism protects cells from oxidative stress by reinforcing cell membranes to limit oxygen availability (7). Interleukin-17 (IL-17)-producing T helper 17 (Th17) cells fulfill an essential role in immune induction and mediation of tissue-resident homeostasis (8). In the intestine, Th17 cells contribute to maintaining the integrity of the intestinal barrier (9) and are implicated in oxidative stress generated by imbalanced oxidative phosphorylation (OXPHOS) (8). As the last intermediate in cholesterol biosynthesis, DES may have alternative effects to cholesterol due to their similar molecular structure. Accordingly, besides involving cholesterol-mediated oxidative stress and inflammatory responses, DES also functions as an endogenous ligand for Th17-targeted key transcription factor RORγt (10). Therefore, the effects of DES on intestinal cell inflammation and oxidative stress pathways deserve further attention.

To explore the molecular regulation actions in the gut, we investigated the potential of DES in core genes involved in the inflammatory response and oxidative stress in the porcine ileum epithelial cells (IPI-2I). We isolated and extracted DES from red macroalgae to generate the natural compounds for cell treatment. The transcriptomic analysis and molecular biological validations were used to evaluate the transcriptional modulation of DES in IPI-2I. Pigs are biomedical models for humans owing to the similarities in physiology and metabolism (11). Thus, our study would provide new insights for further understanding DES physiological functions to benefit human intestinal health through anti-oxidation or immunotherapy.

## 2 Materials and methods

### 2.1 Samples, chemicals, and standards

For sample preparation, the amounts of crude powders (500 g) of macroalgae were sieved and placed into a conical flask, and 95% ethanol was added. Then, the mixed solution was extracted by ultrasound at 60°C for 1 h. Continuous extraction and concentration until ethanol is wholly volatilized. Next, the concentrated extract was successively extracted with an equal volume of petroleum ether, dichloromethane, ethyl acetate, and n-butanol. Further, the organic phase of ethyl acetate was collected and concentrated for DES extraction.

All solvents and reagents were analytical grade or better: 95% ethanol, petroleum ether, dichloromethane, ethyl acetate, n-butanol, methanol, formic acid, acetonitrile, and DES standards (GlpBio, Shanghai, China). The stock solution concentration was calculated considering the purity of commercial standards. Work standard solutions were prepared from the stock solution and diluted with methanol before analysis. Stock solutions containing 1 mL of ethyl acetate were prepared in HPLC-grade methanol. Linear calibration curves (y=189476x+24442) were obtained in the tested concentration ranges for the samples.

### 2.2 LC-MS evaluation of DES content in ethyl acetate extraction solution

DES extractions from macroalgae were determined by a triple quadrupole mass spectrometer LCMS-8050 (Shimadzu, Kyoto, Japan). HSS T3 analytical columns (2.1 mm × 50 mm, 1.8 µm) were used by chromatographic separation, along with 0.4 mL/min flow rate at 40°C. Formic acid in water (0.1%, v/v, solvent A) and acetonitrile (solvent B) were performed as the mobile phase. Solvent A gradient of 0.5 min 25% solvent B, 2 min 25–95% solvent B, 1 min 95% solvent B, 0.1 min 95–25% solvent B, and 2.4 min 25% solvent B was used. The optimized mass parameters: nebulizing gas flow (3 L/min), drying gas flow (15 L/min), interface voltage (3.5 kV), collision-induced dissociation argon gas pressure (270 kPa), desolvation line temperature (250°C), and heat block temperature (400°C). The mass transition for DES was set as m/z 383.25 > 113.20 (-).

### 2.3 Cell culture and cell counting experiment

IPI-2I is obtained from the European Collection of Authenticated Cell Cultures (ECACC). IPI-2I cells were maintained in regular RPMI-1640 medium (Hyclone, UT, USA) supplemented with 10% FBS (Gbico, NY, USA) and 100 mg/mL penicillin-streptomycin (Solarbio, Beijing, China) at 37°C in a 5% CO_2_ humidified atmosphere.

IPI-2I cells were seeded in the 12-well culture plates at a density of 1.5 × 10^5^ cells/well for 12 h and divided into the vehicle group and DES group. The concentration of 5 μM/10 μM DES or DMSO was treated in the indicated wells for another 72 h. The viable cell numbers were counted at 0, 24, 48, and 72 h with a hemocytometer chamber under the microscope.

### 2.4 Cell counting kit-8 assay

To further assess cell viability, cells were seeded in 96-well culture plates at approximately 5 × 10^3^ cells/well in 100 μL of the medium. After 3 days of indicated treatment, 10 μL CCK-8 solution (Dojindo Molecular Technologies Inc., Kumamoto, Japan) and 90 μL Opti-MEM (Gbico, NY, USA) were added to each well with incubation at 37°C for 3 h. Then, a multimode microplate reader determined the absorbance at 450 nm (Spark™ 10M, Tecan GmbH, Austria).

### 2.5 Real-time quantitative PCR

Total RNA extracted from IPI-2I cells using TRIzol Reagent (Takara Biotech, Dalian, China) was reverse-transcribed into cDNA using HiScript^®^ II Q Select R.T. SuperMix (Vazyme, Nanjing, China) according to the manufacturer’s instructions and previous report (12, 13). qRT-PCR analysis was performed by an ABI StepOne Plus Real-Time PCR System (Applied Biosystems, CA, USA) using AceQ^®^ qPCR SYBR Green Master Mix (Vazyme, Nanjing, China). The sequences of primers are exhibited in **Table 1**. The results of relative gene expression were normalized to *GAPDH* and were calculated using the 2^−ΔΔCT^ method.

**Table 1:** Real-time PCR primer sequences.

Name	Primer sequences (5’-3’)	Products Length(bp)
*IL-1β*	F: AAGAAAGTGCGGCGGAAAGTA R: CCACAGAAGTCCCATCCTTAC	177
*IL-6*	F: ATCTGGGTTCAATCAGGAGACCT R: ATTTGTGGTGGGGTTAGGGG	208
*TNF-α*	F: CCTACTGCACTTCGAGGTTATC R: GCATACCCACTCTGCCATT	158
*IFN-γ*	F: CAGCTTTGCGTGACTTTGTG R: GATGAGTTCACTGATGGCTTT	381
*CAT*	F: GCTGGTTAATGCGAGTGGAGAGG R: GGGAAAGTCGTGCTGCGTCTTC	101
*SQLE*	F: ATGTGGACCTTTCTCGGCATTGC R: GGTAGCGACAGCGGTAGGACAG	145
*LRP1*	F: TCTACCACCAGCGGCGTCAG R: CAGCAGGCAGATGTCAGAGCAG	95
*STAT3*	F: TGGAGAAGGACATCAGCGGTAAGAC R: AGGTAGACCAGCGGAGACACAAG	148
*NOD1*	F: GACAACTTGCTGCACAACGACTAC R: ACGAAGAACTCCGACACCTCCTC	137
*RORC*	F: CAATGGAAGTGGTGCTGGTCAGG R: GGGAGCGGGAGAAGTCAAAGATG	150
*GAPDH*	F: ACATCATCCCTGCTTCTACTGG R: CTCGGACGCCTGCTTCAC	187

### 2.6 RNA-seq analysis

Total RNA was extracted from the IPI-2I cells in the vehicle and DES groups. The concentration of RNA was measured with a NanoDrop 2000 spectrophotometer (Thermofisher Scientific, CA, USA), and its quality was evaluated with an Agilent Bioanalyzer 2100 system (Agilent Technologies, CA, USA). The RNA‐seq libraries were constructed using Illumina Tru-Seq RNA Sample Prep Kit (Illumina, CA, USA). The libraries were deeply sequenced using an Illumina HiSeq 2000 sequencer at BGI Tech (Wuhan, China), according to the manufacturer’s instructions. Clean reads with higher quality were aligned to Sscrofa11.1 using TopHat2. For subsequent analysis, the cufflinks software was performed to obtain the quantitative fragments per kilobase of exon model per million mapped fragments (FPKM) values. DESeq 2 software was utilized to perform differential expression of genes between the DES and vehicle groups. The differentially expressed threshold for genes was set as |Log_2_(fold change) | > 1 and adjusted *P* < 0.05.

### 2.7 Kyoto encyclopedia of genes and genomes and gene ontology analysis

Gene Set Enrichment Analysis (GSEA 4.1.0) software was used to identify GO terms enriched in differentially expressed genes (DEGs). Furthermore, statistically enriched biological processes or pathways of DEGs were ranked and classified by the Metascape database (http://metascape.org/) for GO and KEGG pathways. KEGG pathway plot, Volcano plot, and Venn diagram were plotted by an online platform for data analysis and visualization (http://www.bioinformatics.com.cn).

### 2.8 Western blot assay

The cells were seeded in 6-well culture plates and treated as described above (vehicle and DES groups). After washing thrice with cold PBS, cells were lysed on ice with 300 μL RIPA buffer (Beyotime, Shanghai, China) containing protease inhibitors. Cellular proteins were obtained by centrifugation at 12000 × *g* for 10 min at 4°C and determined using the BCA Protein Assay Kit (CWBiotech, Beijing, China). Proteins were separated in 8-10% SDS-PAGE gels and transferred onto PVDF membranes (Millipore, MA, USA). The membranes were blocked with 5% skimming milk and incubated with RORγ primary antibody (Invitrogen, MA, USA, 14-6988-82, 1:1000) and GAPDH primary antibody (Proteintech Ltd, Wuhan, China, 10494-1-AP, 1:1000) overnight at 4°C. Then the membranes were incubated with HRP-conjugated secondary antibodies. Finally, the membranes were visualized with an Enhanced ECL Chemiluminescent Detection kit (Vazyme, Nanjing, China) using the automatic chemiluminescence imaging analysis system (Tanon, Shanghai, China). The relative integrated density was normalized against GAPDH expression. Western blot bands were quantified using the Image J software.

### 2.9 ELISA detection

The concentrations of pro-inflammatory cytokines (IL-1β, IL-6, TNF-α, and IFN-γ) in the cell supernatant were determined using porcine ELISA kits (Solarbio, Beijing, China) according to the manufacturer’s instructions.

### 2.10 Reactive oxygen species determination

Intracellular ROS abundance was determined by the ROS assay kit (Solarbio, Beijing, China). After the DES treatment, the cells were incubated with DCFH-DA probes at 37°C for 30 min, according to the manufacturer’s instructions. Thereafter, the collected cells were measured using a microplate reader (Spark^TM^ 10M, Tecan GmbH, Austria). The relative fluorescence intensity (RFI) was measured at 488/525 nm.

### 2.11 Statistical analysis

Statistical analysis was performed with GraphPad Prism 8.0 software by Student’s *t*-test to compare the means, and data were shown as mean ± SD. The differences were considered significant at *P* < 0.05. All figures were displayed with GraphPad Prism 8.0 software. All data were repeated at least 3 times.

## 3 Results

### 3.1 Linear calibration curves construction and contents detection

**Figure 1:**
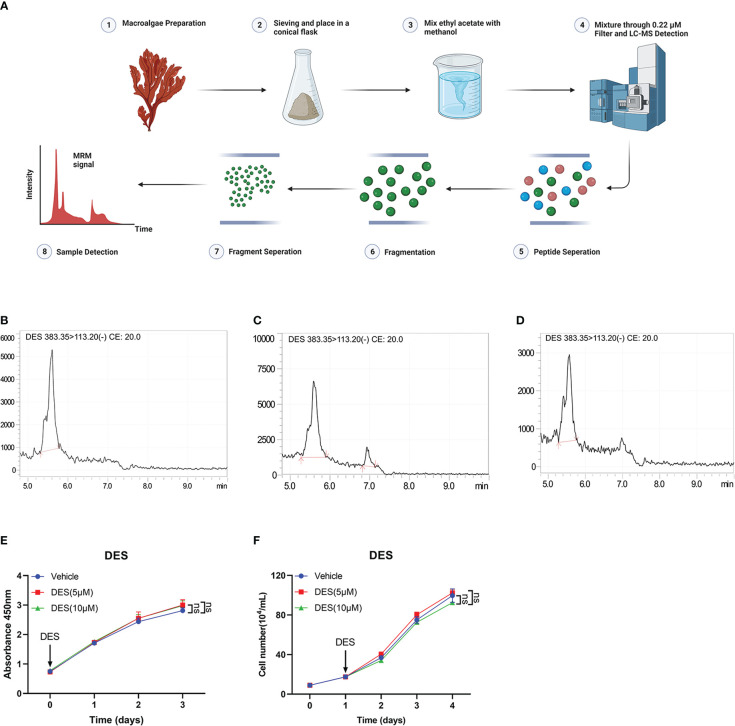
DES does not affect cell growth and survival in IPI-2I cells. **(A)** Overview of sample preparation and detection workflow. **(B)** DES was determined by LC-MS and quantified with multiple reaction monitoring (MRM) mode. MRM chromatograms of DES standards in 250 μg/mL **(C)** and 25 μg/mL **(D)** determined by LC-MS. **(E)** DES (5 μM/10 μM) treatment in IPI-2I cells for 0, 24, 48, and 72 h by CCK-8 detection. **(F)** Cell counting analysis at the indicated time (0, 24, 48, and 72 h) with DES (5 μM/10 μM) treatments in IPI-2I. The data are shown as the means ± SD, n ≥ 3 per group. The experiments were repeated 3 times. ns, represents differences not significant, using Student’s *t*-test analysis.

In this study, LC-MS analysis and MRM workflow were performed to quantify the contents of DES extractions (**Figures 1A, B**). An internal standard calibration curve was constructed with standard solutions of DES ranging from 0.025 to 0.25 μg/mL. As shown in **Figures 1C, D**, the linear regression equation y=189476x+24442 was used to determine the content of DES in the pretreatment sample as 125 μg/mL. These results provide the processes of DES extraction and content measurement.

### 3.2 DES does not affect cell growth and survival in IPI-2I cells

To investigate the protective function of DES in intestinal epithelial cells, cell proliferation assay was performed in IPI-2I and IPEC-J2 cells with different concentrations of DES (0, 2.5, 5, and 10 μM). The result of cell viability showed that DES treatment had no effects on cell proliferation in IPI-2I at the indicated time (0, 24, 48, and 72 h), compared to that in the vehicle (**Figure 1E**). Consistently, DES also had no effects on the cell counting analysis ranging from 0 to 72 h (**Figure 1F**). Similarly, the effects of DES on IPI-2I and IPEC-J2 were further evidenced by the inconspicuous changes in cell morphology and cell number (**Supplementary Figures 1A, B**). These results demonstrated that DES does maintain the physiology of the intestinal epithelial cells.

### 3.3 DES drives inflammatory response and alleviates oxidative stress

**Figure 2:**
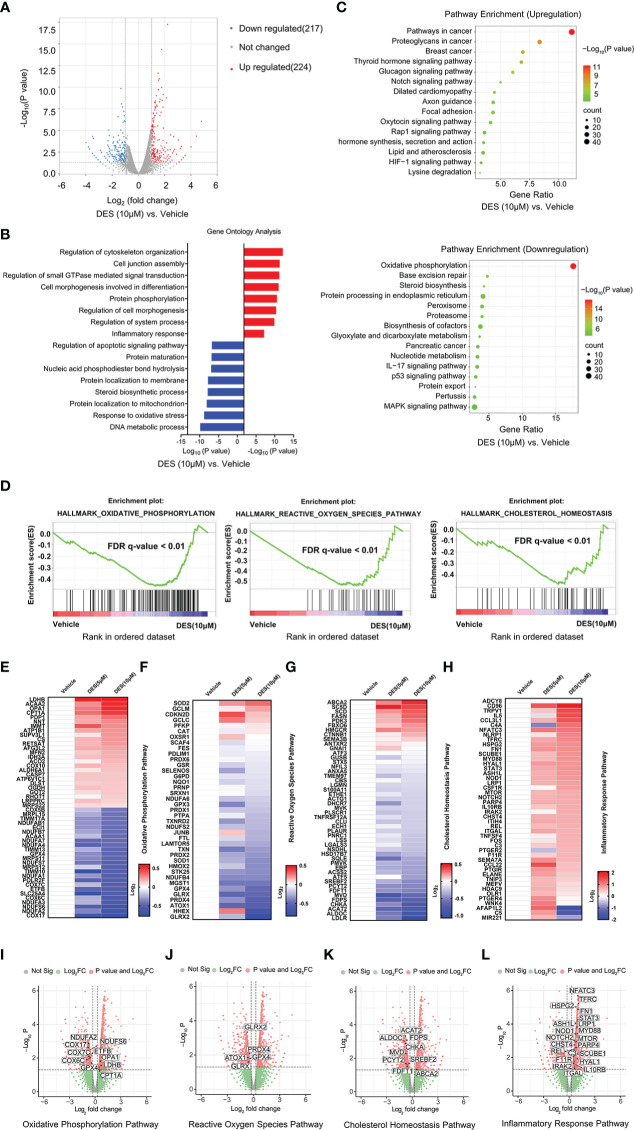
DES drives inflammatory response and alleviates oxidative stress pathways in IPI-2I cells. **(A)** Volcano plot visualization of the differential gene expression profiles between the DES (10 μM) and vehicle group by transcriptome analysis. **(B)** Genes expression involved in the inflammatory response pathway were among the most enriched pathways analyzed by GO. **(C)** DEGs involved in the OXPHOS pathway were the most abundant downregulated enrichments analyzed by the KEGG. **(D)** The GSEA depicting the enrichment of DEGs downregulated in the cholesterol homeostasis, OXPHOS, and ROS pathways from DES (10 μM) versus vehicle in IPI-2I. FDR, false-discovery rate. **(E–H)** Heatmaps of mRNA expression (RNA-seq, Log_2_ transformed) changes of the aforementioned **(B–D)** pathways. **(I–L)** Volcano plot visualization of DEGs in the aforementioned **(B–D)** pathways from DES (10 μM) versus vehicle in IPI-2I.

To identify the key transcriptional pathway regulated by DES, transcriptome analysis was performed using the IPI-2I cells treated with or without DES (5 μM/10 μM). In total, we identified 441 DEGs (|Log_2_(fold change) |> 1, *P* < 0.05) between DES (10 μM) and vehicle groups, comprising 224 upregulated and 217 downregulated genes (**Figure 2A**, **Table S1**). Further function annotations of transcripts are shown in **Figures 2B, C** and **Table S2**. The GO and KEGG pathway enrichment analysis of DEGs revealed that genes were most enriched in the inflammatory response and OXPHOS pathways. Further analysis by GSEA also demonstrated that the signatures involving OXPHOS, ROS, and cholesterol homeostasis pathways were strongly downregulated by DES (**Figure 2D**, **Table S3**). In association with the GO and KEGG pathway enrichment, the pathway-focused genes subset indicated that a vast majority of the OXPHOS, inflammatory response, and cholesterol homeostasis pathways were significantly altered (**Figures 2E–L**, **Table S4**). It indicated their roles in response to the pro-inflammatory and anti-oxidative stress effects of DES. Intriguingly, the DES (5 μM) treatment showed a similar alteration of these pathways (**Supplementary Figures 2A–E**, **Tables S5**–**8**). Collectively, these findings indicated that inflammatory response and oxidative stress regulated by DES treatment might be the predominant processes in IPI-2I cells.

### 3.4 RORγ acts as a key factor to regulate inflammatory response and ROS

**Figure 3:**
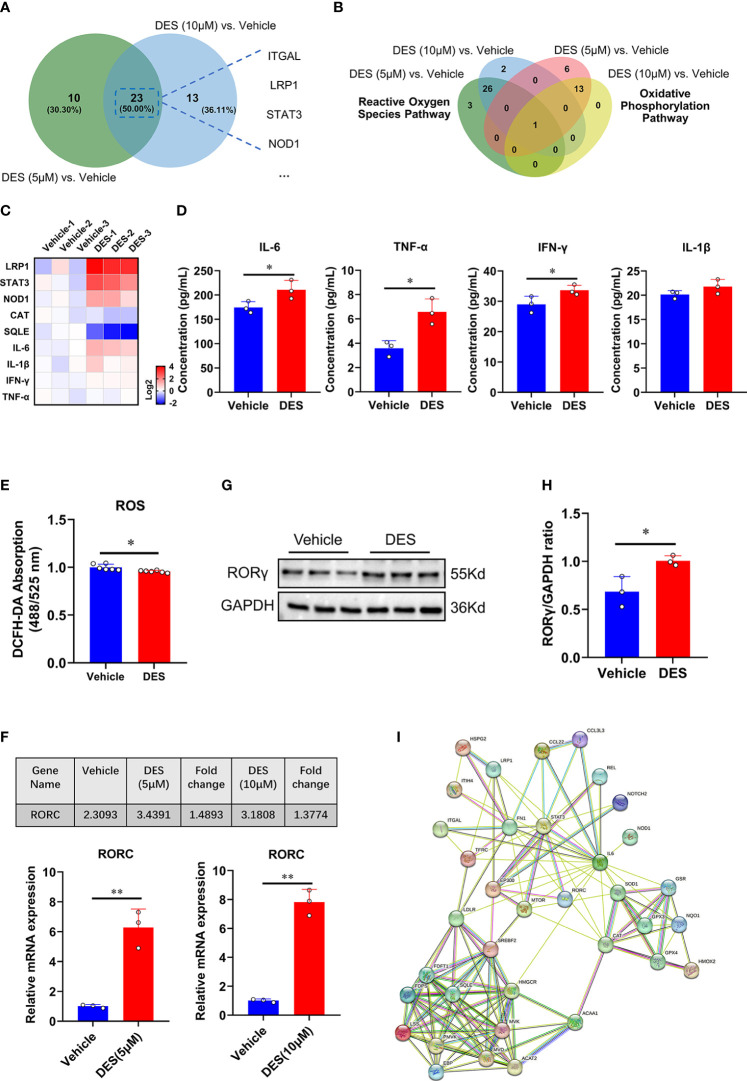
RORγ acts as a potential factor to regulate inflammatory response and oxidative stress pathways. **(A)** Venn diagram of the genes with significantly differential expression (Log_2_ fold change > 0.5) shared by DES (5 μM) versus vehicle and DES (10 μM) versus vehicle in inflammatory response pathway. **(B)** Venn diagram of the genes with significantly differential expression (Log_2_ fold change < -0.5) shared by DES (5 μM) versus vehicle and DES (10 μM) versus vehicle in ROS and OXPHOS pathways. **(C)** A heatmap shows fold change (in Log_2_) of 9 genes determined by qRT-PCR analysis in IPI-2I treated with DES (10 μM) for 72 h **(D)** Protein levels of IL-6, TNF-α, IFN-γ, and IL-1β in the cell supernatant. **(E)** Measurement of ROS (DCFH-DA) fluorescence abundances with DES (10 μM) treatment. **(F)** RNA-seq (FPKM value) and qRT-PCR analysis of *RORC* gene expression by DES (5 μM/10 μM) treatment. **(G)** Western blot analysis of RORγ protein expression in the DES (10 μM) group. **(H)** The relative protein expression of RORγ was normalized to the GAPDH. **(I)** The interactions among inflammatory response, ROS, and cholesterol homeostasis pathway key proteins involved in RORγ transcriptional regulation were predicted by STRING-ELIXIR analysis. The data are shown as the means ± SD, n ≥ 3 per group. The experiments were repeated 3 times. *P < 0.05, **P < 0.01, using Student’s *t*-test analysis.

Having shown the potential pathway enrichment of DES in the IPI-2I cells, we further explored which DEGs exert critical roles in the pathways mentioned above. Accordingly, 23 upregulated DEGs in the inflammatory response pathway were identified with DES (5 μM/10 μM) supplementation in the Venn diagram (**Figure 3A**). Moreover, a critical anti-oxidative stress gene *GPX4* involved in both OXPHOS and ROS pathways was highly enriched with DES (5 μM/10 μM) addition (**Figure 3B**). To further validate the expression pattern of DEGs, 9 genes (*LRP1*, *STAT3*, *NOD1*, *IL-6*, *TNF-α*, *IFN-γ*, *IL-1β*, *CAT*, *SQLE*) were quantified by qRT-PCR (**Figure 3C**). Similarly, the expression patterns of detected genes showed a high concordance with differential analysis results of RNA-seq. In line with the mRNA expression, pro-inflammatory cytokines (IL-6, TNF-α, and IFN-γ) in the supernatant were also significantly elevated by the DES treatment (**Figure 3D**, *P* < 0.05). In contrast, ROS abundance was significantly reduced by DES (10 μM) (**Figure 3E**). Notably, nuclear receptor RORγ is a promising therapeutic target of the inflammatory response and has a mechanistic link with oxidative stress. We found that the mRNA (**Figure 3F**) and protein expressions (**Figures 3G, H**) of RORγ were upregulated by DES. Interestingly, STRING-ELIXIR analysis demonstrated that the putative transcriptional activators STAT3, IL-6, and GPX4 interacted with RORγ (**Figure 3I**, **Table S9**). Taken together, these results suggest that DES promotes the RORγ pivotal regulation associated with genes involved in the inflammatory response and oxidative stress.

## 4 Discussion

**Figure 4:**
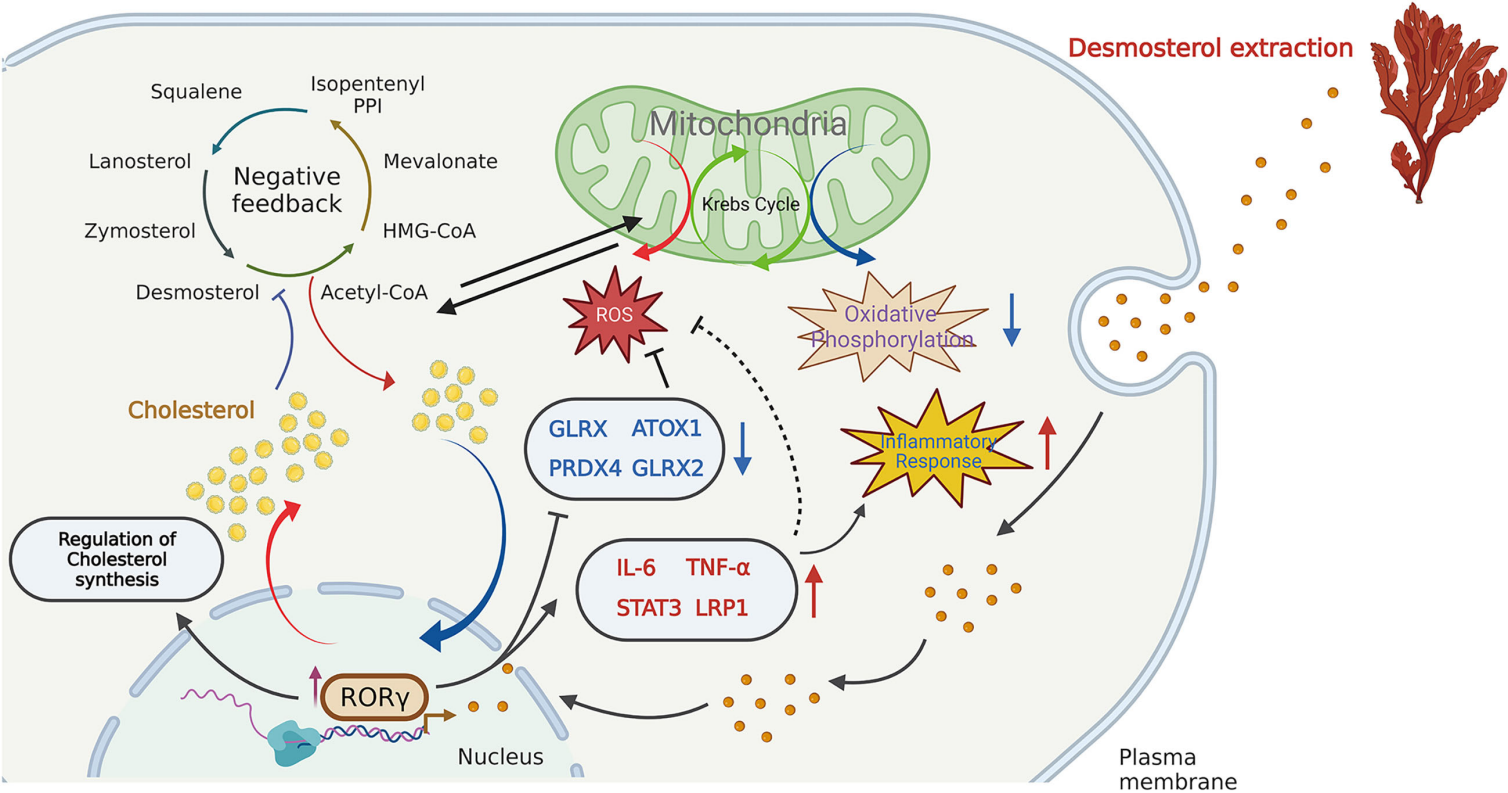
Schematic illustration of the DES-mediated transcriptional regulation in cholesterol biosynthesis, pro-inflammatory and oxidative stress. DES causes the highly expressed RORγ in the IPI-2I and facilitates cholesterol production. The increased cholesterol inhibits the endogenous cholesterol *de novo* synthesis in a negative feedback loop. Again, DES exerts anti- oxidative effects by downregulating oxidative stress- regulated gene expression (*GLRX*, *PRDX4*, *ATOX1*, *GLRX2*, etc.). Moreover, DES also upregulated the expression of inflammation-related genes (*IL-6*, *TNF-α*, *STAT3*, *LRP1*, etc.) to activate the immune response.

In recent years, increasing attention has been devoted to the influences of inflammation and immune regulation by cholesterol metabolism. Three essential possibilities have been proposed to explain the cholesterol potential roles: (1) as an important precursor to steroid hormones that regulate immune response (14); (2) as an endogenous intermediate in the bile acids conversion to activate innate immune signaling (15); (3) as metabolites in bile acids that regulate their derivatives (isoallolithocholic acid) on differentiation of anti-inflammatory regulatory T cells (Treg) (16). As described by Hu et al. (10) and Santori et al. (17), cholesterol precursor (DES) has been proven to bind to RORγ and directly regulate its immunoactivity in Th17 cells. In the present study, we analyzed the main regulatory effects of DES in porcine intestinal epithelial cells, involving cholesterol homeostasis, RORγ expression, OXPHOS, ROS, and inflammatory response pathways. A graphic illustration of the DES-mediated transcriptional regulation of DES in pro-inflammatory and oxidative stress is the process shown in **Figure 4**. RORγ, an orphan nuclear receptor, can directly bind to intermediates of cholesterol biosynthesis or interact with SREBP2 to facilitate cholesterol synthesis (18, 19). Meanwhile, as a nuclear hormone receptor, the activity of RORγ is also influenced and tightly regulated by endogenous ligands (20). Here we show that DES administration significantly increases RORγ expression in IPI-2I cells. Upregulated expression of RORγ further causes the activation of endogenous cholesterol synthesis. Indeed, increased cholesterol contents lead to the inhibition of OXPHOS pathways. Moreover, RORγ expression improves pro-inflammatory cytokine expression and attenuates ROS abundance by interacting with anti-oxidative genes. Therefore, a logical hypothesis will be that by DES co-option effectively enforce their immunity-activation response and anti-oxidative stress program with activated RORγ in IPI-2I cells.

The role of cytokines has been implicated in both maintaining homeostasis and inflammatory intestinal disorders (21). Among them, pro-inflammatory cytokines as classical regulators that modulate inflammatory responses and facilitate intestinal homeostasis (22). In the present study, we found that cell supernatant concentration of IL-6, TNF-α, and IFN-γ was increased by DES treatment. Notably, apart from producing the classical cytokines in response to inflammation, DES also mediates inflammatory response by activating some non-typical genes (such as *STAT3*, *NOD1*, and *LRP1*) expression. Elevated IL-6 levels are observed in inflammatory processes, stimulating the JAK/STAT3 signaling hyperactivation and inducing immunosuppression (23). Furthermore, the NOD1/NF-κB signaling pathway is activated by LPS to produce the pro-inflammatory cytokines, resulting in inflammation (24). LRP1 can reduce oxidative stress-induced apoptosis to alleviate pathological damage (25). We observed that these factors involved in the inflammatory response pathway are upregulated following DES treatment. It may be a protective response to exogenous stimuli. Indeed, CYP27A1-27hydroxycholesterol-modulated reduction of cholesterol density inhibits the activation of IL6-JAK-STAT signaling pathway (26). Our results further provide evidence that the genes enriched in the inflammatory response are related to oxidative stress and cholesterol homeostasis pathways in the STRING-ELIXIR analysis. Increased expression of RORγ may result from Th17 cell differentiation, which enhances defensive inflammation response. The different cytokines facilitate activated T-cell differentiation into various lineages of effectors (10). In agreement with this notion, Tregs are also crucial for immune tolerance and homeostasis (27). There is evidence that the depletion of Treg can provoke and enhance immune response (27). However, other regulation processes could be beyond the pathways mentioned above, which is a limitation and warrants further investigation.

The influences of marine algae extract, such as amino acids, fatty acids, polyphenolic compounds, and vitamins, on inflammation and ROS have already been reported in different models (28–30). We found that DES, a red algae extract, can also function as a pro-inflammatory and anti-oxidant scavenger. This versatility and capability to act directly or indirectly to improve immunity make natural DES extract highly appealing for nutraceutical development. As safe and healthy products, DES can enhance disease resistance and has excellent application prospects for humans and animals. Next, we would focus on toxicity evaluation, and safety validation of DES extract in animals to refine the overall exploration of its function. It is noteworthy that we also have provided a reliable extraction method for both marine algae and other sterols.

Collectively, supplementation of the DES provides a set of negative feedback signals leading to changes in cholesterol metabolism and then action on the inflammatory processes, immune response, and oxidative stress. DES-triggered biological events are probably *via* the activation of RORγ-mediated transcription. Although primarily descriptive, we provide evidence that DES is a natural candidate for nutraceutical and health product development.

## Data availability statement

The data presents in the study are deposited in the NCBI Sequence Read Archive repository, and the accession number is SRR22526287, SRR22526288, and SRR22526289, respectively.

## Author contributions

DC and WB conceived the study; HQ and QZ performed most of the experiments; HW and SW participated in the experiments; HQ, QZ, H-YL, and DC participated in its design and coordination and wrote the manuscript. All authors contributed to the article and approved the submitted version.
