# Supplementary material for: Desmosterol: A natural product derived from macroalgae modulates inflammatory response and oxidative stress pathways in intestinal epithelial cells
Source: Front Immunol. 2023 Jan 4;13:1101643. doi: 10.3389/fimmu.2022.1101643 (PMC9845693; doi:10.3389/fimmu.2022.1101643)
Supplement: Supplementary file 1 [file DataSheet_1.docx]

Supplementary Material

## Supplementary Figures


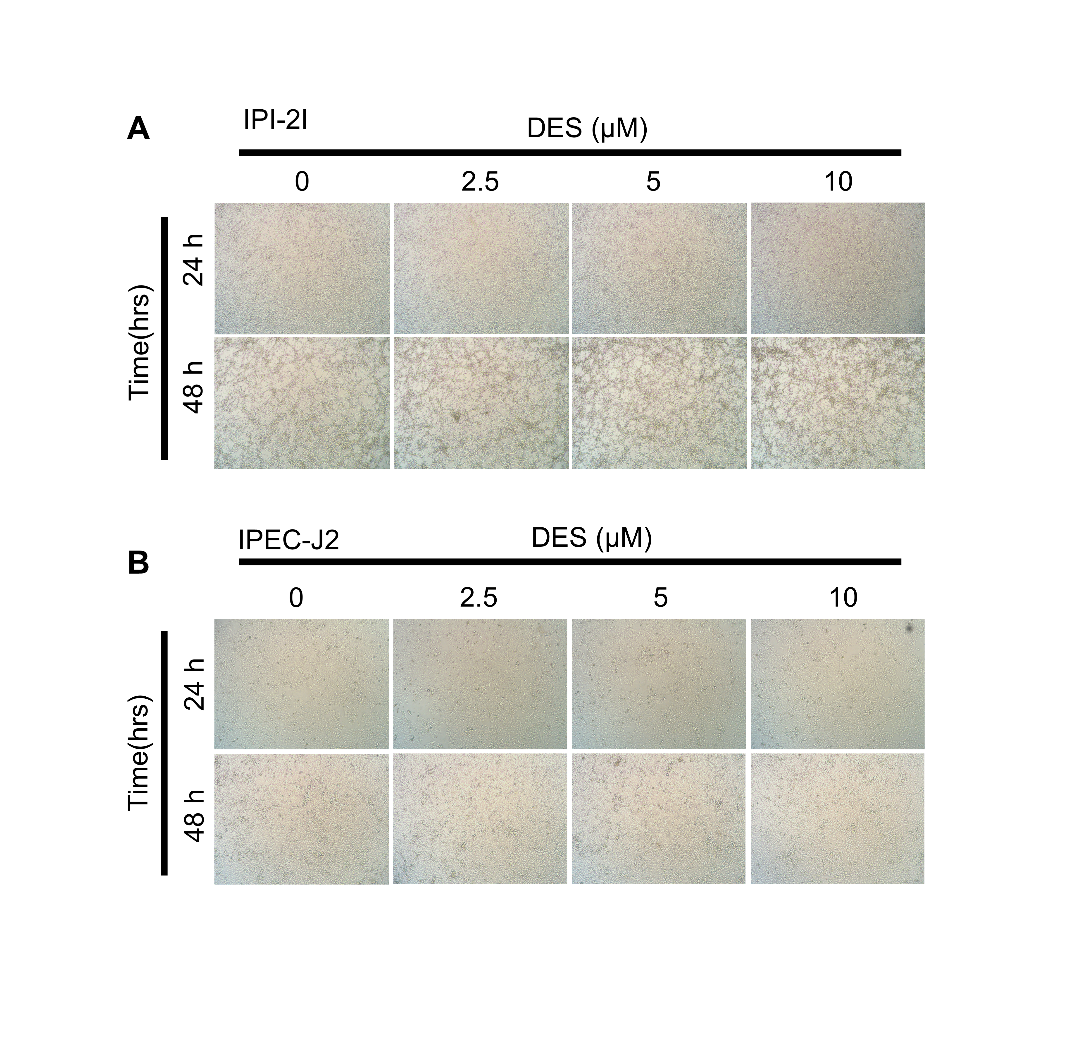


Supplementary Figure 1 **DES does not affect cell morphology and cell number.** **(A)** DES (2.5 μM/5 μM/10 μM) treatment for 24 h and 48 h in IPI-2I cells. **(B)** DES (2.5 μM/5 μM/10 μM) treatment for 24 h and 48 h in IPEC-J2 cells. Cell morphology and cell density were captured (40 × magnification) under microscope.


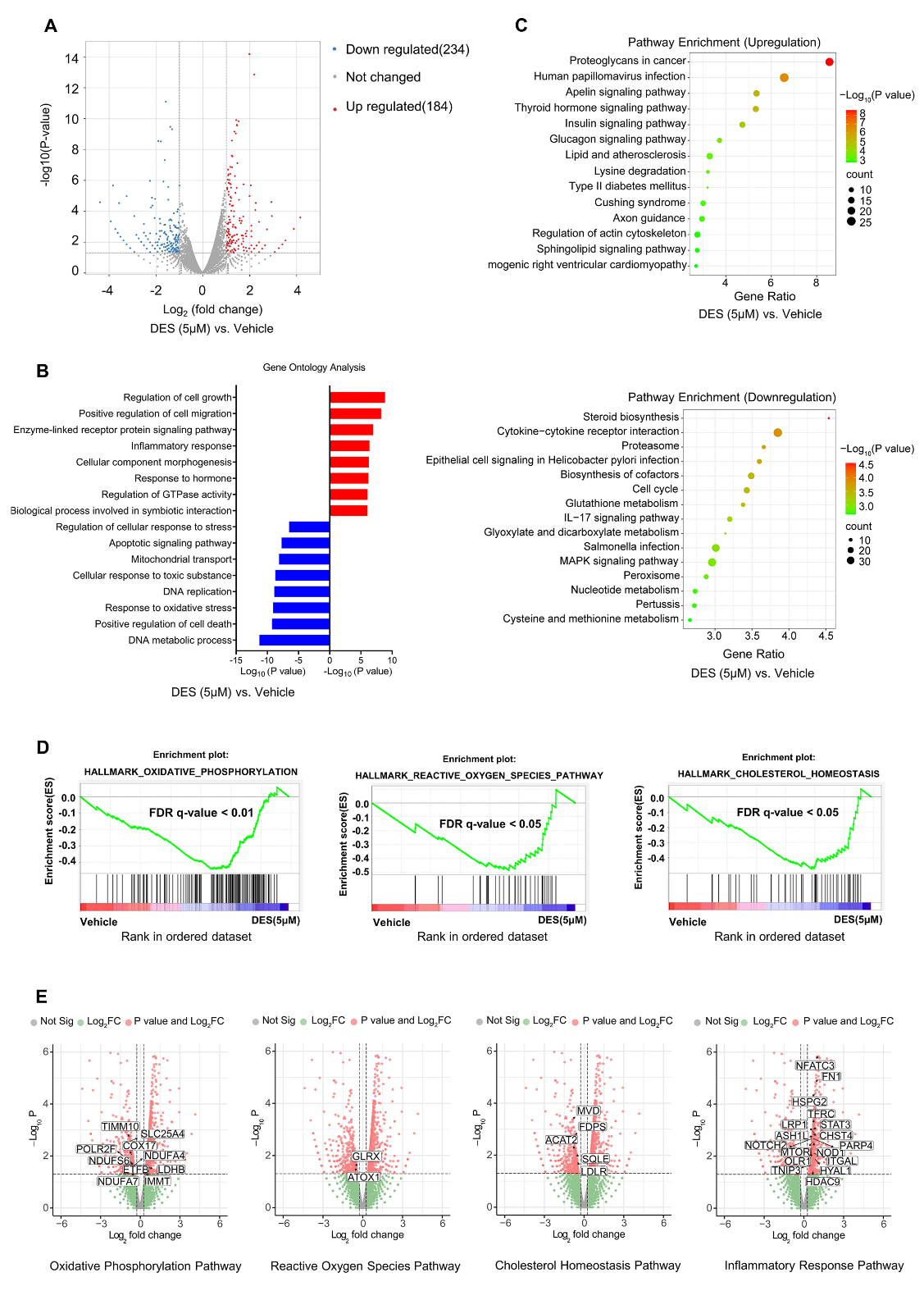


Supplementary Figure 2 **Enrichment of inflammatory response and oxidative stress pathways by DES administration in IPI-2I cells. (A)** Volcano plot visualization of the differential gene expression profiles between the DES (5 μM) and vehicle group by transcriptome analysis. **(B)** Genes expression involved in the inflammatory response and oxidative stress pathways were among the most enriched pathways analyzed by GO. **(C)** DEGs involved in the steroid biosynthesis pathway were the most abundant enrichments analyzed by KEGG. **(D)** The GSEA depicting the enrichment of DEGs downregulated in the cholesterol homeostasis, OXPHOS and ROS pathways from DES (5 μM) versus vehicle in IPI-2I. FDR, false-discovery rate. (E) Volcano plot visualization of DEGs in the aforementioned (B-D) pathways from DES (5 μM) versus vehicle in IPI-2I.
